# Effect of Acetic Acid and Sodium Bicarbonate Supplemented to Drinking Water on Water Quality, Growth Performance, Organ Weights, Cecal Traits and Hematological Parameters of Young Broilers

**DOI:** 10.3390/ani11071865

**Published:** 2021-06-23

**Authors:** Yordan Martínez, Cristopher Isaac Almendares, Cristhian José Hernández, Mavir Carolina Avellaneda, Ana Melissa Urquía, Manuel Valdivié

**Affiliations:** 1 Poultry Research and Teaching Center, Agricultural Science and Production Department, Zamorano University, P.O. Box 93, San Antonio de Oriente 11101, Honduras; cristopher.almendare.2020@alumni.zamorano.edu (C.I.A.); cristhian.hernandez.2020@alumni.zamorano.edu (C.J.H.); 2 Plant Pathology, Diagnosis and Molecular Research Lab, Agricultural Sciences and Production Department, Zamorano University, P.O. Box 93, San Antonio de Oriente 11101, Honduras; cavellaneda@zamorano.edu; 3 Environment and Development Department, Zamorano University, P.O. Box 93, San Antonio de Oriente 11101, Honduras; aurquia@zamorano.edu; 4 National Center for Laboratory Animal Production, P.O. Box 6240, Santiago de las Vegas, Rancho Boyeros 10100, La Habana, Cuba; mvaldivie@ica.co.cu

**Keywords:** blood indicator, broiler, cecal characteristic, visceral and immune organ, water pH

## Abstract

**Simple Summary:**

Currently, many poultry productions supply drinking water extracted from wells or other sources whose physical, chemical and microbiological characteristics are sometimes unknown, especially pH and its impact on production and health of the birds during their productive life. The following study evaluated the effect of an acidifier (acetic acid 0.4%; T1), an apparently neutral pH (T2) and the use of an alkalizing (sodium bicarbonate 1%; T3) in drinking water on growth of broilers during their first 10 days of life. T3 changed the normal parameters of drinking water for the broilers, and this treatment also provoked high mortality, ascites and poor productive development, and it modified the relative weight of the immune organs, liver, heart and pancreas, as well as the cecal pH, although cecal lactic acid bacteria count and hematological indicators were unchanged. Likewise, it is contradictory that T1 did not show improvements in performance and cecal traits compared to drinking water with an apparently neutral pH. Therefore, the supply of alkaline water (due to high Na content) affects the performance, health, immunity and digestive physiology of broilers, which shows that the control of this parameter in drinking water has a direct impact on animal growth and the economy of the poultry producer.

**Abstract:**

To evaluate the effect of acetic acid and sodium bicarbonate supplemented to drinking water on water quality, growth performance, relative organ weights, cecal traits and hematological parameters of broilers, a total of 456 one-day-old Cobb MV × Cobb 500 FF mixed broilers were randomly placed in three experimental treatments, with four replicates per treatment and 38 birds per replicate, for 10 days. The treatments consisted of the use of acetic acid (0.4%; T1) as acidifier, an apparently neutral pH (T2) and sodium bicarbonate (1%; T3) as alkalizer of the drinking water. T3 showed the highest values (*p* < 0.05) for total dissolved solids, electrical conductivity, salinity and pH. T1 and T2 showed the same productive response (*p* > 0.05); however, T3 decreased (*p* < 0.05) body weight, feed intake and the relative weight of the pancreas and immune organs and increased (*p* < 0.05) water intake, mortality and relative weight of the heart and liver. Likewise, T3 increased (*p* < 0.05) the cecal pH, although without changes for the cecal lactic cecal bacteria count and blood parameters (*p* > 0.05). The acid pH of the drinking water had no effect on the biological response of broilers compared to T2; however, the T3 provoked high mortality, ascites, low productivity and abnormal growth of some organs.

## 1. Introduction

The post-hatching stage of broilers has many critical issues to consider. Throughout their first 10 days of life, broilers undergo a series of critical transitions that affect the way they receive nutrients; therefore, bird care and observation during this period are important for optimal flock performance [1]. Newborn chicks need to consume feed and water immediately after hatching to supply energy requirements and hydrate the body for various metabolic functions, respectively [2]. Therefore, the percentage of broiler viability is higher when the consumption and quality of feed and water is adequately controlled [3].

Approximately 70% of the total bird weight is water; therefore, broilers should consume at least twice as much water compared to feed, being the main vehicle of the feed bolus in the gastrointestinal tract (GIT) [4]. Therefore, water quality from any source requires exhaustive control, mainly in physical–chemical parameters such as turbidity, electrical conductivity, total dissolved solids, hardness, total chlorine and pH; and biological parameters such as the presence of pathogens, since all of these directly influence water consumption and animal performance. Water pH has been related to impact on digestive and immune organs and poultry health [5]; thus, Engberg et al. [6] mentioned that the pH within each segment of the gastrointestinal tract determines the chemical environment and directly influences the digestion and absorption of nutrients. Although the animal organism has the ability to buffer intra and extracellular pH, some results show that the acidic drinking water supplied in the initial stage of fattening broilers reduces pH in some parts of the gastrointestinal tract, such as the jejunum, ileum and cecum, which promotes a decrease of pathogenic bacteria colonization [7]. Similarly, Hajati [8] mentioned that acidic drinking water provides a second layer of protection against lactic acid bacteria, which allows its establishment as part of the ecology of the enteric system.

Thus, the use of organic acids to lower drinking water pH is a common practice in many poultry companies, especially in periods of stress; however, the scientific results are inconsistent. In this sense, Bouassi et al. [9] and Açıkgöz et al. [10] reported improvements in growth performance when they used organic acids on drinking water in poultry. However, Hamid et al. [11] and Martinez et al. [12] did not find productive benefits in broilers when acidifying the water with organic acids, and some animals also presented metabolic disturbances. On the other hand, few studies have been developed to know the effect of alkaline drinking water on broiler production, considering that many sources of water used by poultry farms have high Na content [13]. In this sense, Klasing [14] reported that an excess of Na in the drinking water of broilers causes dehydration, heart failure with edema, ascites and high mortality. The comparative study of different pHs of drinking water in broilers will allow us to know its direct effect on the digestive system, immune organs, productivity, cecal microflora and possible conditions in the most critical stage (the first 10 days of life) of this poultry category. The present study evaluated the effect of acetic acid and sodium bicarbonate supplemented to drinking water on water quality, growth performance, visceral and immune weight organs, cecal traits and hematological parameters of apparently healthy young broilers.

## 2. Materials and Methods

The Science and Agricultural Production Department at Zamorano University, located in San Antonio de Oriente (Honduras), reviewed and approved all the standardized procedures performed in this experiment, which were conducted under the Guidelines for Experimental Animals (Reference number: 20245 and 20193).

### 2.1. Study Location and Site

The study was developed at the Poultry Research and Training Center of Zamorano University, located in Valle del Yeguare at km 32 of the Tegucigalpa–Danlí highway, Honduras. The training and research center has an average temperature of 26 °C, at an altitude of 800 m above sea level, and an annual rainfall of 1100 mm.

### 2.2. Animals and Treatments

A total of 456 one-day-old Cobb MV × Cobb 500 FF mixed broilers were collected equally, 50% male and 50% female from same flock. The broilers were randomly placed in three experimental treatments, four replicates per treatment and 38 birds per replicate, for 10 days. It should be noted that this trial was carried out in the starter phase of broilers (the first 10 days of age), which is considered the most critical productive stage in the growth of these animals [2,15,16,17]. Additionally, a diet was formulated considering the nutritional requirements of the genetic line under study (Table 1) [18]. The treatments consisted of the use of acetic acid (0.4%; T1) as acidifier, an apparently neutral pH (T2) and sodium bicarbonate (1%; T3) as alkalizer of the drinking water [12]. The allowed limits of water pH (6.5–8.5) [5] were considered to acidify and alkalize the drinking water supplied to broilers.

### 2.3. Experimental Conditions

Each replica consisted of a bed of wooden chip in each pen and with a density of 10 birds/m^2^. The supply water was ad libitum using plastic gallon waterers, as was the feed mash in plastic hopper feeders. Temperature and ventilation inside the shed were controlled by gas breeders, curtain handling and fans. The photoperiod distribution was as follows: 0–7 days old, 23 light (L):1 dark (D); 8–10 days, 20 L:4D [18]. The shed was disinfected according to environmental quality standards of Poultry Research and Training Center Protocol, 24 h before the chicks entered the experimental area, this was disinfected with quaternary ammonium (5%). No medicines or therapeutic veterinary care were used throughout the experimental stage. Birds were vaccinated against Newcastle disease, Gumboro disease and bronchitis on their first day.

### 2.4. Drinking Water Quality

Water quality parameters including temperature (°C), pH, electrical conductivity (EC), total dissolved solids (TDS) and salinity (SALT) were analyzed using a multiparameter meter, model WD-35604-00 (Oakton Electronic, Vernon Hills, IL, USA), with an accuracy of ±0.01 at pH, ±0.5°F/O, and ±1% (full scale) for EC, TDS, and SALT parameters. Additionally, water turbidity was measured using the standardized method of nephelometry with a SperDirect turbidimeter 860040 (Sper scientific, Scottsdale, AZ, USA) with an accuracy of ±5%. Residual chlorine was measured by colorimetry with a CN66-F kit (HACH, Loveland, CO, USA), which has a measuring range of 0.1–3.4 mg/L. Additionally, the presence and/or absence of fecal coliforms was performed by counting on plates using 3M^TM^Petrifilm^TM^ (3M, Saint Paul, MN, USA). All analyses were done five times.

### 2.5. Growth Performance

The indicators of the broiler’s growth performance were determined on a weekly basis. Viability was determined by living animals among those existing at the beginning of the experiment. The feed and water intake were calculated using the offer and reject method. The feed conversion ratio was calculated as the amount of feed ingested, for a gain of 1 kg of body weight. The initial and final individual weight of each stage was taken using a Navigator OHAUS scale, model N38110, with a precision of ±1 g (OHAUS^TM^, Parsippany, NJ, USA).

### 2.6. Relative Weight of Digestive and Lymphoid Organs and Intestinal pH

At 10 days old, 20 chicks per treatment with six hours of fasting were selected randomly and weighed individually before slaughter on a Navigator OHAUS model N38110 scale with a precision of ±1 g (OHAUS^TM^, Parsippany, NJ, USA). The birds were euthanized using the mechanical cervical dislocation method; the procedure was performed by a certified veterinarian. The following organs were extracted and weighed: immune organs (bursa of Fabricius, spleen and thymus), and digestive organs (cecum, gizzard, proventriculus and small intestine). The organs were weighed using a BLAZE BL scale, model 100-01-BK, with a precision of ±0.01 g (Dalman Enterprises Ltd., Wycombe, Buckinghamshire, UK). After slaughter, the small intestine and caecum pH of 10 broilers per treatment were determined by a digital potentiometer Oakton^®^ model 700 digital pH meter (Oakton Instruments, Vermon Hills, IL, USA). Following the manufacturer’s recommendations, the potentiometer was calibrated using pH buffers of 1.68, 4.01, 7.00, 10.01 and 12.45.

Moreover, the left cecum of five birds per treatment were randomly selected. The mucosa was scraped with a scalpel for microbiological culture. The cecal content of each sample was placed in a sterilized tube, and the weight was recorded and diluted with Butterfield’s phosphate buffered dilution water to a 1:9 ratio (weight:volume). The diluted cecal contents were homogenized, and serial dilutions (1/10) were made from it until achieving a 10^5^ dilution. Aliquots of 0.1 mL of each dilution were spread onto the surface of a plate with MRS agar (Neogen Acumedia, Lansing, MI, USA) supplemented with methylene blue (0.016 g/1000 mL) with a pH of 5.6, for 48 h at 37 °C in anaerobiosis (Gas Pak^TM^ BBL, Cockeysville, MD, USA). Counts of lactic acid bacteria were reported as log 10 CFU/g by colonies’ morphology on MRS + MB agar. Gram stain and catalase activity were tested on each type of the reported colony [19]. A Labomed model LX400 light microscope (Labomed Inc., Los Angeles, CA, USA) was used to characterize bacterial morphology. All the microbiological tests were performed in the Food Microbiology Laboratory of the Zamorano University.

### 2.7. Hemogram

On day 10, a hematological examination was performed on six fasting broilers per treatment. The blood was removed (10 mL) by puncture of the left-wing vein and deposited in tubes with anticoagulants. In blood plasma, erythrocytes and leukocytes were determined by automatic counting in a Neubauer Chamber model 68058-15 (Electron, Microscopy, Sciences, Hatfield, PA, USA) using 2B methyl violet as a diluent. Additionally, platelets were quantified in the Neubauer Chamber model 68058-15 (Electron, Microscopy, Sciences, Hatfield, PA, USA) using ammonium oxalate solution. The differential leukocyte formula was determined by peripheral blood smear (PSF), with Wright’s stain, and observed in the microscope with a 100× objective for the differentiation of heterophiles, eosinophils, lymphocytes, monocytes and basophils. The hematocrit–CVP was determined by the capillary microcentrifugation process. Hemoglobin was determined according to the hemotest method. Mean corpuscular volume (MCV) and mean corpuscular hemoglobin (MCH) are determined according to the formulas: MCV (fl) = (Hct (L/L)/RBC (10^12^/L)) × 1000 and MCH = Hb (g/L)/RBC (millions/µL).

### 2.8. Statistical Analysis

The data was processed using a one-way analysis of variance (ANOVA) in a completely randomized design; before carrying out the analysis of variance, the normality of the data was verified using the Kolmogorov–Smirnov test, and the Bartlett’s test was used to evaluate the uniformity of the variance, where necessary. Finally, Duncan’s test was used to determine the differences between means (*p* < 0.05). All the analyses were performed using the statistics software SPSS version 23.0 (SPSS Inc., IBM Corporation, New York, NY, USA).

## 3. Results

### 3.1. Water Quality

Table 2 shows the physical, chemical and microbiological characteristics of drinking water for broilers. Throughout the three treatments, pH, electrical conductivity and total dissolved solid count showed significant differences (*p* < 0.05) among each other, whereas salinity in T3 was higher (*p* < 0.05) than in T1 and T2. However, the temperature showed no differences (*p* > 0.05) among treatments. The presence of total and fecal coliforms was not found. In addition, T1 and T3 had no presence of residual chlorine in relation to neutral water.

### 3.2. Growth Performance

**Figure 1:**
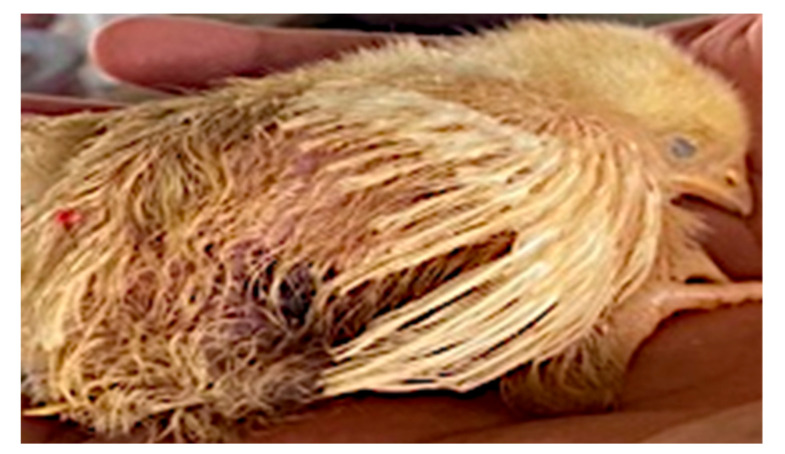
Broiler with growth retardation in T3.

The effect of acetic acid and sodium bicarbonate on drinking water on growth performance of broiler chickens (0–10 days) is shown in Table 3. T3 decreased body weight and feed intake and increased mortality, feed conversion ratio and water intake compared to other treatments (*p* < 0.05). However, both treatments (T1 and T2) did not show statistical differences between them for any measured indicators (*p* > 0.05). Additionally, Figure 1 indicates that the broilers consuming water with sodium bicarbonate showed growth retardation, and at 10 days old, all the slaughtered broilers had ascites (Photo 2), but this was not so for the other experimental treatments.

### 3.3. Relative Organ Weights, Intestinal pH and Cecal Lactic Acid Bacteria

The relative weight of the proventriculus, gizzard, small intestine and cecum showed no noticeable differences among treatments (*p* > 0.05; Table 4). However, T3 increased (*p* < 0.05) the relative weight of the heart and liver and decreased (*p* < 0.05) the relative weight of the thymus and spleen compared to the other two treatments (Table 4). In addition, this treatment (T3) decreased (*p* < 0.05) the pancreas relative weight in relation to T2. In addition, the relative weight of bursa of Fabricius decreased (*p* < 0.05) due to the acidification (acetic acid, T1) and alkalization (sodium bicarbonate, T3) of the drinking water (T3). Likewise, the Table 4 indicated that T3 increased the cecum pH with significant differences with the other experimental groups (*p* < 0.05). However, the small intestine pH and cecal lactic acid bacteria count showed no differences (*p* > 0.05) among treatments.

### 3.4. Hemogram

The results of the hemogram presented in Table 5 showed that the acetic acid and sodium bicarbonate used as acidifying and alkalizing agent, respectively, did not modify (*p* > 0.05) any hematological parameter of young broilers.

## 4. Discussion

One of the goals of this study was to determine whether use of acetic acid as acidifier and sodium bicarbonate as alkalizer in drinking water had an influence on the water quality, which could explain its effect on the biological response of young broilers. In this sense, Vermilion et al. [20] have reported that the immune system benefits from acidic water because it mimics the natural pH of the crop, though alkaline drinking water weakens humoral immunity and limits the genetic potential of broilers. However, the use of acidifiers and alkalizers in drinking water also has a direct influence on other indicators of water quality. In this sense, the drinking water supplied to broilers should not contain excessive levels of minerals [21]; thus, when the electrical conductivity exceeds 3000 µS/cm, it causes a decrease in palatability and an increase in diarrheal syndrome [22]. Based on the results of this study, it is observed that the T1 and T2 did not exceed the aforementioned parameters; however, the T3 showed 4800 µS/cm, much higher than the permissible limit for this indicator (electrical conductivity). Likewise, Oh et al. [23] mentioned that electrical conductivity is an indicator of the degree of mineralization (total ion concentration) of the water. Therefore, the electrical conductivity is related to the salinity and concentration of the total dissolved solids.

Moreover, Cobb-Vantress [24] defined that values between 3000 and 4999 ppm of total dissolved solids could engender aqueous feces, increasing mortality rate and slowing down growth. In this study, T3 recorded 3443 ppm, which could directly affect the animal response. Additionally, Aviagen [25] determined that calcium, magnesium and sodium salts are the primary components that contribute to high of TDS, which are pollutants commonly responsible for altering productive performance, specifically increasing mortality rate. This study used 1% sodium bicarbonate to alkalize the drinking water; thus, an excess level of sodium in feed or water increases blood volume, which requires more cardiac work in broilers; increased blood flow or increased resistance to flow in the lungs results in right ventricular hypertrophy, increased venous pressure and ascites [21]. The relationship between these three parameters (total dissolved solids, salinity and electrical conductivity) was demonstrated in this experiment, where T3 showed a higher concentration of electrical conductivity, total dissolved solids and salinity (Table 2). Therefore, T1 and T2 are considered adequate for intake, while T3 could slow down the development process of broilers and immune system weaken [21,22,23].

Furthermore, both treatments that modified the pH of drinking water (with sodium bicarbonate and acetic acid) totally reduced residual chlorine (Table 2); apparently, changes in the pH directly influence the self-decomposition of chlorine [26]. In this sense, the World Health Organization [27] informed that chlorination of water is not effective with pH values higher than 7.2 and lower than 6.8. Thus, this test did not detect residual chlorine in drinking water with a pH of 4.01 and 8.60 (Table 3). Additionally, Len et al. [26] found the total loss of residual chlorine when the water had a pH lower than 2.5 and higher than 9.0. It should be noted that the addition of acetic acid and sodium bicarbonate in the drinking water did not influence the water temperature, turbidity, total coliforms and fecal coliforms (Table 2).

At present, many scientific works have demonstrated the effect of organic acids as acidifiers of drinking water in the production of broilers, although the results are not consistent due to several factors such as the environment, feed palatability, buffer capacity of the diet, concentration of organic acid used, management, gut health, presence of other antimicrobial compounds, water pH and genetic expression of poultry [28], and many poultry companies continue to frequently use these organic acids (mainly acetic acid) in drinking water, mainly in stressful situations, with the aim of reducing the colonization of intestinal pathogenic bacteria and promoting competitive exclusion [12].

In this sense, Mohammed et al. [29] have shown that the use of acetic acid in drinking water improved the growth performance and the health condition of young broilers. Moreover, Ocampo et al. [30] showed that acidic water in the early stages in broilers reduced the mortality in relation to the control group. Additionally, Ahmad et al. [31] reported positive results on growth performance of broilers with heat stress when they used KCL as an acidifier in drinking water. Furthermore, Chaveerach et al. [32] and Wolfenden et al. [33] concluded that acidified drinking water could prevent the growth of *Campylobacter* spp. and *Salmonella enteritidis* in broiler flocks, respectively.

However, in this study, T1 and T2 did not indicate notable differences for the main productive indicators of broilers (Table 3). Similar results were obtained by Martínez et al. [12], who did not find an improvement in performance and viability; these authors concluded that organic acids have a better productive response when broilers are exposed to different challenging conditions. Both treatment (T1 and T2) showed a similar and low mortality (0.65%; Table 3); perhaps under the experimental conditions of this study, the acidic pH of the drinking water is not necessary until 10 days.

On the other hand, it is clearly observed that T3 depressed the body weight, feed intake, conversion feed ratio and mortality in 17.09, 15.38, 5.65 and 13.82%, respectively, compared to T2 (Table 3; Figure 1). However, Chung et al. [34] indicated contrary results when they used alkaline drinking water (8.05) from magnetization in the initial phase of broilers. In this sense, El-Sabrout and Hanafy [35] found that magnetized water had higher concentrations of salinity, total hardness, Na^+^, K^−^, Ca^2+^, Mg^2+^, Cl_−_, HCO_3_^−^ and pH compared to tap water. The aim of water magnetism is that the magnetic field increases the surface tension of the water with an increase in the water pH and the shear viscosity, which inhibits the formation of scale [36]. Although more reliable studies are needed to understand how magnetized water with an alkaline pH might be recommended in the poultry industry, it appears that the effect of alkaline pH in broilers will be mediated by the concentration of electrolytes in the water. According to Sayed and Downing [37], an optimal balance of electrolytes in diet formulation and drinking water for broilers regulate blood and fluid retention. Therefore, excess sodium in the diet or drinking water causes hypernatremia, which increases fluid retention, blood flow, resistance to flow and water consumption for fluid balance [38], a finding found in this study (Table 3).

The results obtained (Table 3) in this study agree with Rojas et al. [39] and Farfán et al. [40] who reported a higher intake of this liquid (water) when using high concentrations of sodium bicarbonate in the drinking water of broilers. In this sense, Olanrewaju et al. [41] stated that the increase in the sodium concentration in water increases the extracellular volume, causing a loss of intracellular water, which is offset by an increase in fluid intake. Additionally, Ahmad et al. [42] found a higher water intake and a better productive response in broilers with heat stress when they used three different sodium salts, namely sodium bicarbonate (NaHCO_3_), sodium carbonate (Na_2_CO_3_) and sodium sulphate (Na_2_SO_4_), which demonstrates that the vehicle (feed and water) for sodium bicarbonate supplementation will have a direct impact on the animal response. Undoubtedly, the results obtained in this study are a consequence of an increase in electrolyte consumption in broilers.

**Figure 2:**
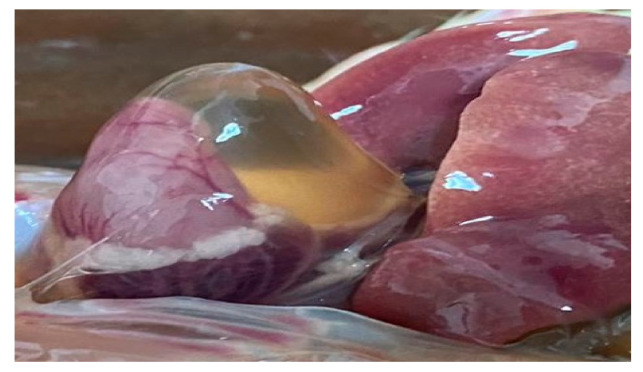
Broiler with ascites in T3.

In this regard, the necropsies performed on broilers (10 days old) that consumed alkaline water due to the use of sodium bicarbonate showed evidence of accumulation of water in the abdominal area (ascites) in 100% of the animals (Figure 2), which could influence the high mortality recorded in broilers (14%). The results obtained in this study are supported by Ahmadipour et al. [43], who maintains that high ppm of TDS triggers ascites in broilers. The ascites syndrome is characterized by right congestive heart failure, with generalized hydrostatic venous hypertension, right heart hypertrophy and edema [44]. Currently, improvements in genetics and diet have decreased the incidence of ascites syndrome; however, T3, with an increase in electrical conductivity, total dissolved solids and salinity (Table 2), provoked this pathological condition in broilers [24,25]. It should be noted that this study could contribute to understanding the incidence of ascites syndrome in poultry units that use brackish drinking water from wells, mainly near the coast.

Interestingly the experimental treatments did not affect the relative weight of the proventriculus, gizzard, small intestine and cecum (Table 4). However, this study showed that the sodium bicarbonate used as an alkalizer also directly affected the relative weight of the visceral and immune organs of broilers. In this sense, T3 increased the relative weight of the heart, liver and pancreas (Table 4) due to ascites syndrome and hypernatremia; thus, one of the most representative symptoms is cardiac hypertrophy [44] due to venous hypertension and intracellular water loss, which provokes higher functionality of the organ. In this sense, Klasing [14] indicated that a sodium concentration higher than 0.04% (4000 ppm) induces toxicity, ascites syndrome and cardiomegaly, the latter sign caused by cardiac weakening, dilation and hypertrophy. In addition, the intake of electrolytes (mainly sodium) changes the acid–base balance, causing temporary changes in all fluid compartments [45]. Several strategies have been proposed to reduce the incidence of ascites and improve the development of internal organs and animal production, such as early dietary restriction, lower density of diets, restriction of access time to feed and modification of the growth of chickens [46].

In addition, hypernatremia, in this experiment, due to excess Na in drinking water, could cause hepatomegaly in broilers due to the formation of fibrinous exudate in the blood vessels, which allows the exit of large molecules rich in fibrinogen, forming a fibrin network [47]. In this sense, Martínez et al. [2] found a positive correlation between the relative weight of the heart, liver and spleen due to hepatic portal blood circulation, and between the pancreas and liver due to homeostatic regulation for insulin and glucagon secretion [48], which helps maintain stable glucose levels, which is why these organs were also affected due to the T3. Therefore, this shows that the use of sodium bicarbonate and pH alkaline in drinking water appears to have three characteristic symptoms: hepatomegaly, cardiomegaly and pancreatomegaly in the first 10 days of life in broilers.

It is important to note that the use of acetic acid and acidic pH of the drinking water had no effect on the relative weight of these organs (Table 4). In this sense, Abdel-Fattah et al. [49] found no notable changes in relative gizzard weight when lactic, acetic and citric acid were used in broiler diets. However, Martínez et al. [12] found variations in the relative weight of proventriculus, gizzard, small intestine and liver when they used acetic acid in the drinking water of broilers, although the authors found no changes in the relative weight of the cecums. Furthermore, Mohammadi et al. [50] indicated that organic acids statistically modified the relative weight of the liver in broilers. This shows that the relative weight of the digestive organs in apparently healthy animals, as was the case with the T1 and T2, will depend on several intrinsic and extrinsic factors such as the physiological and immunological status of the broilers, age, diet, technology and stressful situations.

Although there are contradictions in the interpretation of the variations in the relative weight of the immune organs and the productive response in broilers, many authors have related better immunological activity with the relative weight of the bursa of Fabricius, thymus and spleen of these animals [51]. It is clearly observed that the T3 depressed the relative weight of these lymphoid organs (Table 4), which could influence the high mortality of this treatment (Table 3); thus, it was observed that the relative weight of the thymus decreased in 0.14% (Table 4). This organ is taken as an indicator of the bird’s health status because it acts in situations of chronic stress, being responsible for the differentiation and development of T lymphocytes [7]. Additionally, interestingly, the bursa of Fabricius, which is the organ responsible for the maturation and differentiation of B lymphocytes [49,52], decreased its relative weight with T1 and T3 by 0.05 and 0.02%, respectively (Table 4), which could elucidate that both drastic changes in water pH could reduce the immunological specificity of this organ. However, other experiments are necessary to demonstrate this hypothesis. Additionally, Maxwell et al. [53] informed that ascites syndrome decreases the relative weight and the activity of the bursa of Fabricius due to congestive heart failure. However, Fascina et al. [28] did not record significant differences (*p* > 0.05) in the relative weight of immune organs when they used organic acids and neutral water in broiler production.

One of the main justifications for the use of organic acids, especially acetic acid, in drinking water is that they can lower cecal pH and reduce the growth of pathogenic bacteria in critical stages and under various stressors in broilers [54]. In this sense, Dibner and Butin [55] recommended the use of organic acids to lower intestinal pH, improve nutrient absorption, and decrease the incidence of infections in broilers. In this sense, Hamid et al. [11] showed that ingestion of acidified water lowered the pH of the gastrointestinal tract and improved productivity in broilers compared to apparently neutral water. Likewise, Martínez et al. [12] showed that the use of organic acids in drinking water decreased the cecal pH but did not lower the pH of the small intestine. Likewise, Jaramillo [7] registered a significant decrease in cecal pH when supplemented with organic acids and antibiotics.

However, our study showed that despite supplying drinking water with a pH of 4.1 (Table 2), this did not cause a decrease in cecal pH in broilers (Table 4); similar results were obtained by Abdelrazek et al. [56] when they found no positive results when they used acetic acid in broilers. In this sense, Santos et al. [57] have mentioned that these natural products have a higher effect on the gastrointestinal tract when animals are subjected to stressful conditions. The main contradictions of the effect of organic acids on intestinal pH are related to the buffering capacity of dietary ingredients, presence of other antimicrobial compounds, heterogeneity of gut microbiota and the production conditions [58,59]. Apparently, the experimental conditions of this study were adequate considering the performance and the cecal pH of the birds in the groups that consumed apparently neutral and acidic water. Additionally, the diets intentionally did not have growth-promoting antibiotics. On the other hand, it is clearly observed that the significant increase in the cecal pH of the broilers was due to the alkaline drinking water (Table 4). Rynsburger and Classen [58] argue that high pH increases protein denaturation and pepsin activity in young broilers, influencing nutrient uptake and animal growth, something that occurred in this experiment (Table 3), as these animals had the worst productive performance.

The cecal microbiota is known to be abundant in lactic acid bacteria such as *Lactobacillus*, *Pediococcus, Oenococcus* and *Leuconostoc*, which directly influence gut health, nutrient absorption and the productive response of broilers [59]. Some authors have related the growth of cecal lactic acid bacteria and the decrease in the pH of this organ, because it favors competitive exclusion and intestinal health [60]. However, Martínez et al. [12], showed that acetic acid in drinking water reduced the lactic bacteria count due to an excessive decrease in cecal pH, something that did not occur in this experiment, since the pH remained unchanged when drinking water was supplied with a pH of 4.01 (Table 4). In this sense, Agboola et al. [61] did not find notable changes in the lactic acid bacteria count in any gut section when they used organic and probiotic acids on broiler diets. Other studies with natural products also found no changes in pH and cecal lactic acid bacteria in broilers [19], which shows that acetic acid in these apparently healthy broilers has no effect on the total count of cecal lactic acid bacteria. Interestingly, the T3 that caused high mortality and notable changes in the relative weight of the immune and visceral organs (Table 3 and Table 4) and the cecal pH of broilers had no effect on the cecal lactic acid bacteria count (Table 4). Although few studies have indicated the effect of sodium bicarbonate on cecal lactic acid bacteria, it appears that the GIT buffer system and its acid–base balance contributed to the cecal pH being in the normal ranges for young broilers [2], despite the fact that the addition of sodium bicarbonate to drinking water could cause metabolic alkalosis as a consequence of the increase in alkalis in the blood [62]. Furthermore, these results demonstrate that T3 has a more marked effect on metabolism and immunity than on the cecal microflora of young birds. However, other studies are necessary to verify these hypotheses.

At present, hematological parameters are used as indicators of health in humans and animals. Variations in these indicators can reflect parasitic, fungal, viral and bacterial infections as well as intoxication, dehydration or blood clotting problems [63]. This study showed that although the experimental treatments (mainly T3) changed the growth performance, cecal pH and the relative weight of some digestive and immune organs (Table 3 and Table 4), they did not provoke changes on the hemogram of broilers up to 10 days old (Table 5). Nosrati et al. [64], Nguyen et al. [65] and Gilani et al. [66] found similar results when including acidifiers in drinking water and in the feed of broilers; although they found changes in blood biochemistry, performance and gut health, it seems that acidifiers do not induce an abnormal immune response in broilers. Other non-enzymatic natural products can cause changes in polymorphonuclear cells (neutrophils and eosinophils), with the aim of eliminating exogenous material and/or possible toxic and allergenic compounds [67].

On the other hand, alkaline water, possible hypernatremia and ascites syndrome did not cause significant changes in these blood parameters in broilers (Table 5). In this sense, Farfán et al. [40] found no notable differences in hematocrit, hemoglobin, plasma protein, white blood cells and red blood cells when they used sodium bicarbonate, ammonium chloride and sodium chloride treatments in heat stress in broilers to prevent respiratory alkalosis. Additionally, it is important to note that multiple intrinsic and extrinsic factors can cause ascites syndrome in broilers; hypoxia, temperature and the presence of pathogenic bacteria have been described as the most predominant extrinsic causes [68,69,70]. According to Maxwell et al. [53] the ascites syndrome in young broilers is characterized by an increase in hemoglobin and hematocrit, although heterophilia, monocytosis, lymphocytosis and decreased blood volume are observed depending on the triggering factors. Ascites in broilers caused by hypernatremia is poorly studied; thus, this experiment in young birds (up to 10 days old) may contribute to understanding the effect of other non-infectious etiologies that induce ascites syndrome.

## 5. Conclusions

The use of acetic acid as an acidifier in the drinking water had no effect on the biological response of the young chickens (up to 10 days old) compared to an apparently neutral pH in the drinking water. However, the use of sodium bicarbonate and therefore an alkaline pH and high values of salinity, electrical conductivity and dissolved solids in drinking water provoked high mortality, ascites, low productive efficiency, abnormal growth of the heart and liver and low activity of the immune organs, although without effects on the digestive organs, cecal count of lactic acid bacteria and blood count of these animals.

## Figures and Tables

**Table 1:** Ingredients and nutritional contributions of broilers diet (0–10 days).

Ingredients	Starter (%) (0–10 Days)
Cornmeal	59.00
Soybean meal	32.4
Premix of minerals and vitamins ^1^	0.50
Sodium chloride	0.35
African palm oil	3.53
Choline	0.05
DL-Methionine	0.34
L-Threonine	0.16
L-Lysine	0.32
Calcium carbonate	1.60
Biofos	1.53
Mycofix plus 5.0 ^®^	0.12
Enzymes Lumis Lbzyme X50 ^®^	0.05
Coccidiostat	0.05
Nutritional contributions	
Metabolizable energy (kcal/kg)	2975
Crude protein	22.00
Neutral fiber detergent	15.22
Crude fiber	3.05
Calcium	0.90
P available	0.45
Lysine	1.22
Methionine + Cystine	0.91
Threonine	0.83
Tryptophan	0.20

^1^ Each kg contains vitamin A 11,550 IU, vitamin D3 4300 IU, vitamin E 27.5 IU, vitamin K3 3.85 mg, vitamin B1 2.75 mg, vitamin B2 9.9 mg, vitamin B6 3.85 mg, vitamin B12 22.0 Mcg, niacin 49.5 mg, pantothenic acid 15.4 mg, folic acid 1.38 mg, biotin 166 mcg; selenium 0.09 mg, iodine 0.18 mg, copper 3.00 mg, iron 36.0 mg, manganese 54.0 mg, zinc 48.0 mg, cobalt 0.12 mg.

**Table 2:** Effect of acetic acid and sodium bicarbonate supplemented to drinking water on its physical, chemical and microbiological characteristics.

	Experimental Treatments				
Items	T1	T2	T3	SEM±	*p*-Value
Temperature (°C)	26.37	26.33	26.27	0.033	0.178
pH	4.01 ^c^	7.03 ^b^	8.60 ^a^	0.025	0.001
Electrical conductivity (S/cm)	166.73 ^b^	86.00 ^c^	4850.00 ^a^	30.655	0.001
Total dissolved solids (ppm)	117.67 ^b^	61.20 ^c^	3443.33 ^a^	44.270	0.001
Salinity (ppm)	0.08 ^b^	0.04 ^b^	2.427 ^a^	0.031	0.001
Turbidity (NTU)	0.00	0.00	0.00	-	-
Residual chlorine (mg/L)	0.00	0.20	0.00	-	-
Total coliforms (CFU)	Absence	Absence	-	-	-
Fecal coliforms (CFU)	Absence	Absence	-	-	-

^a,^^b, c^ Means with different letters in the same row differ at *p* < 0.05. SEM: standard error of the mean; CFU: colony-forming units.

**Table 3:** Effect of acetic acid and sodium bicarbonate supplemented to drinking water on growth performance of young broilers (0–10 days).

	Experimental Treatments				
Items	T1	T2	T3	SEM±	*p*-Value
Body weight (g)	220.91 ^a^	220.69 ^a^	188.47 ^b^	3.696	0.001
Feed intake (g)	204.75 ^a^	205.33 ^a^	177.95 ^b^	2.775	0.001
Feed conversion ratio	1.16 ^b^	1.17 ^b^	1.24 ^a^	0.015	0.012
Mortality (%)	0.65 ^b^	0.65 ^b^	14.47 ^a^	2.653	0.007
Water intake (ml)	557.10 ^b^	531.33 ^b^	843.83 ^a^	31.903	0.001

^a,^^b^ Means with different letters in the same row differ at *p* < 0.05. SEM: standard error of the mean.

**Table 4:** Effect of acetic acid and sodium bicarbonate supplemented to drinking water on relative organ weights, intestinal pH and cecal lactic acid bacteria of young broilers (10 days).

	Experimental Treatments				
Items	T1	T2	T3	SEM±	*p*-Value
Proventriculus (g/kg)	1.53	1.11	1.06	0.291	0.466
Gizzard (g/kg)	5.65	5.92	5.21	0.320	0.303
Small intestine (g/kg)	7.34	8.07	6.95	0.431	0.196
Small intestine pH	6.73	6.72	6.86	0.081	0.403
Cecum (g/kg)	0.91	0.91	1.10	0.089	0.241
Cecal pH	6.27 ^b^	6.32 ^b^	6.63 ^a^	0.050	0.001
Cecal BAL (CFU/g)	1.85	2.11	1.97	0.057	0.054
Heart (g/kg)	0.69 ^b^	0.76 ^b^	0.95 ^a^	0.040	0.001
Liver (g/kg)	3.29 ^b^	3.48 ^b^	4.27 ^a^	0.177	0.001
Pancreas (g/kg)	0.54 ^ab^	0.59 ^a^	0.48 ^b^	0.031	0.045
Bursa of Fabricius (g/kg)	0.17 ^b^	0.22 ^a^	0.19 ^b^	0.018	0.009
Thymus (g/kg)	0.30 ^a^	0.26 ^a^	0.16 ^b^	0.026	0.003
Spleen (g/kg)	0.12 ^a^	0.11 ^a^	0.07 ^b^	0.015	0.004

^a,^^b^ Means with different letters in the same row differ at *p* < 0.05. SEM: standard error of the mean. BAL: lactic acid bacteria.

**Table 5:** Effect of acetic acid and sodium bicarbonate supplemented to drinking water on hematological parameters of young broilers (10 days).

	Experimental Treatments				
Items	T1	T2	T3	SEM±	*p*-Value
Erythrocytes (×10 L)	2.60	3.01	2.71	0.184	0.335
Hemoglobin (g/dL)	8.53	9.95	8.87	0.652	0.342
CVP (%)	26.03	30.05	27.03	1.825	0.337
MVC (fL)	108.13	107.70	109.60	3.899	0.938
MCH (pg)	34.27	35.30	36.40	1.191	0.491
PLT (×10 L)	26.90	35.15	28.70	2.699	0.155
Leukocytes(L)	18,076	17050	21526	263.34	0.494
Heterophiles (%)	27.33	27.50	33.67	6.164	0.723
Eosinophils (%)	3.00	2.50	1.67	1.134	0.717
Lymphocytes (%)	64.67	64.50	60.00	4.951	0.761
Monocytes (%)	5.00	5.50	4.67	0.788	0.763
Basophils (%)	0.00	0.00	0.00	-	-

SEM: standard error of the mean. CVP: central venous pressure; MVC: medium corpuscular volume; MCH: mean corpuscular hemoglobin; PLT: platelets.

## Data Availability

Not applicable.
